# The Survival Outcomes of T1aN0M0 Triple-Negative Breast Cancer With Adjuvant Chemotherapy

**DOI:** 10.3389/fonc.2020.01753

**Published:** 2020-09-30

**Authors:** Wen-Fen Fu, Qing-Xia Chen, Xiao-Xiao Wang, Jie Zhang, Chuan-Gui Song

**Affiliations:** ^1^Department of Breast Surgery, Affiliated Union Hospital, Fujian Medical University, Fuzhou, China; ^2^Department of Burn and Plastic Surgery, Affiliated Longyan First Hospital, Fujian Medical University, Longyan, China; ^3^Department of Gynecology and Obstetrics, Fujian Provincial Maternity and Children Health Hospital, Fuzhou, China

**Keywords:** adjuvant chemotherapy, T1aN0M0, triple-negative breast cancer, breast cancer-specific survival (BCSS), overall survival (OS)

## Abstract

**Purpose:** Triple-negative breast cancer (TNBC) is a subtype with distinct heterogeneity, high invasiveness, and poorer prognosis. There is a controversy about adjuvant chemotherapy (ACT) at the T1aN0M0 stage. This study was carried out to assess the survival benefit of ACT for these patients.

**Methods:** We identified 1,099 patients with T1aN0M0 TNBC who were diagnosed between 2010 and 2016 from the Surveillance, Epidemiology, and End Results (SEER) database. Univariate and multivariable analyses were conducted to determine factors related to survival. One-to-one (1:1) propensity score matching (PSM) was applied to construct a matched sample consisting of pairs of ACT and non-ACT subjects. Breast cancer–specific survival (BCSS) and overall survival (OS) of the two groups were evaluated by Kaplan–Meier plots and Cox proportional hazard regression models. Stratified analysis according to different variables was also performed.

**Results:** No obvious differences in demographic or clinical characteristics were found between patients who had ACT and those without ACT therapy in terms of race, marital status, laterality, or radiation therapy. A higher proportion of patients who were older, had a higher histological grade tumor, and who received breast-conserving surgery had adjuvant chemotherapy. The ACT group did not exhibit better survival in BCSS or OS before PSM. After PSM, the ACT and non-ACT groups consisted of 255 patients, respectively, and Kaplan–Meier curves and multivariate analysis both indicate that adjuvant chemotherapy was not associated with better survival in terms of BCSS or OS. Furthermore, we did not observe any survival advantage in any subgroup irrespective of age, race, marital status, histological grade, surgery type, or radiotherapy status.

**Conclusions:** The study results indicate that there is no strong association between ACT and better survival in T1aN0M0 TNBC. It implies that the chemotherapy decision should be made cautiously and further research into therapeutic strategies are needed in T1aN0M0 TNBC patients.

## Introduction

Triple-negative breast cancers (TNBC), which account for about 10–20% of breast cancer (1), are defined as tumors that lack the expression of estrogen receptor (ER), progesterone receptor (PR), and human epidermal growth factor receptor 2 (HER2) (2). They are characterized by higher invasiveness, heterogeneity, and a relatively poorer outcome than other subtypes (2–4). The clinical outcome of TNBC is associated with age at diagnosis, race, tumor size, histological grade, lymph node status, and the BRCA1 mutation as reported in many studies (1, 5, 6). Unlike other subtypes of breast cancer that receive treatments, such as endocrine therapy or trastuzumab, the main and only current systemic treatment option for patients with TNBC is chemotherapy (2). In response to the enhanced cancer awareness and widespread application of mammography screening, the detection of early stage breast cancer has increased in recent years (7, 8), and small TNBC tumors are not an exception. The appropriate management and treatment of these TNBC patients has received significant attention in recent studies.

The prognosis for patients with small breast cancer tumors is favorable, according to some past reports. Overall survival (OS) for 8 years and relapse-free survival (RFS) for 6.9 years are both more than 90% (9–11). However, patients with HR-negative/HER2-negative tumors had the lowest 5-year distant RFS (DRFS) compared to other subtypes (12). Tumor size is not a strong indicator of prognosis owing to it being highly aggressive and the underlying biological characters being fundamentally different from other breast cancer subtypes (13). Consequently, a reasonable and effective treatment pattern for small-size TNBC tumors, including T1a tumors whose diameter is no more than 0.5 cm, is particularly important. The National Comprehensive Cancer Network (NCCN) and the St. Gallen panel suggest considering ACT for T1bN0M0 TNBC but do not recommend it to T1aN0M0 TNBC patients. Although the latest NCCN guidelines note that some patients with high-risk features, very young women with high-grade histology, for example, may be considered for chemotherapy. Given the lack of evidence, there is still controversy about whether or not to use ACT in patients with T1aN0M0 TNBC. Several retrospective studies state the relationship between disease-free survival (DFS) or OS advantage and chemotherapy in subcentimetric, node-negative TNBC (11, 14–16). Regretfully, the data related to T1a TNBC is limited.

To explore the impact of ACT on breast cancer-specific survival (BCSS) and OS of T1aN0M0 TNBC, this study reviewed T1aN0M0 TNBC cases registered in the Surveillance, Epidemiology, and End Results (SEER) Program of the National Cancer Institute (NCI). SEER is an authoritative source of information on cancer incidence and survival in the United States and currently collects and publishes cancer incidence and survival data from various locations and sources throughout the United States.

## Materials and Methods

### Patients

SEER^*^Stat version 8.3.6 was used to obtain data from the SEER 18 registries research database, which are available for cases diagnosed from 2000 through the current data year. We reviewed 1,148 cases that were primarily sifted from the SEER database with the following conditions: female, year of diagnosis from 2010 to 2016, breast cancer as the first and only malignant cancer, unilateral origin of primary cancer, T1aN0M0 derived from AJCC 7th or 8th edition, and a triple-negative subgroup. Further information included age at diagnosis, race record (white, black, other), marital status at diagnosis, histological grades (I–IV), AJCC staging, laterality, RX Summ-Surg Prim Site (breast-conserving or mastectomy), record of radiation therapy, record of chemotherapy, cause of death classification, and survival in months. There were 35 patients without grade information, and 14 patients without surgery treatment or with an unknown type of surgery who were excluded. Eventually, 1,099 patients were enrolled in this study.

Patients were categorized into two groups according to their ACT treatment status. Patients with an unknown chemotherapy history were classified into the group without ACT. Patients with race of American Indian/AK Native, Asian/Pacific Islander were classified into one group as “others.” The group “not married” consisted of separated, widowed, single (never married), divorced, and unmarried or domestic partner. Breast-conserving surgery (BCS) means removing the gross primary tumor and some of the breast tissue, and other types belong to the mastectomy group. Radiation after surgery, intraoperative radiation, and intraoperative radiation with other radiation before/after surgery were regarded as having radiation therapy. BCSS was defined as the period from the date of diagnosis to the date of death due to breast cancer or the follow-up time if patients were alive, and OS was defined as the duration from the date of diagnosis to the date of death because of all causes (including breast cancer) or the follow-up time.

### Statistical Analysis

Most statistical analysis was conducted utilizing the SPSS version 20.0 software package (IBM SPSS Statistics, Chicago, IL, USA). The baseline characteristics of the included patients were compared between the two groups classified as those receiving chemotherapy and those not receiving chemotherapy using a chi-squared test. A Cox proportional hazards model was utilized to calculate the HR ratio and 95% confidence intervals in the univariate and multivariate analyses and to identify prognostic factors. R program version 3.6.3 and survival package were used to generate the survival curves, and the log-rank test was performed to compare the unadjusted BCSS and OS rates of the two groups of patients. We performed propensity score matching (PSM) by using the psmatch2 module in Stata version 14.0 (Stata Corp., College Station, TX, USA). The command matched each patient with ACT to one patient without ACT using factors including age, race, marital status, histological grade, surgery type, and radiotherapy.

## Results

### Baseline Characteristics of the Study Population

**Figure 1:**
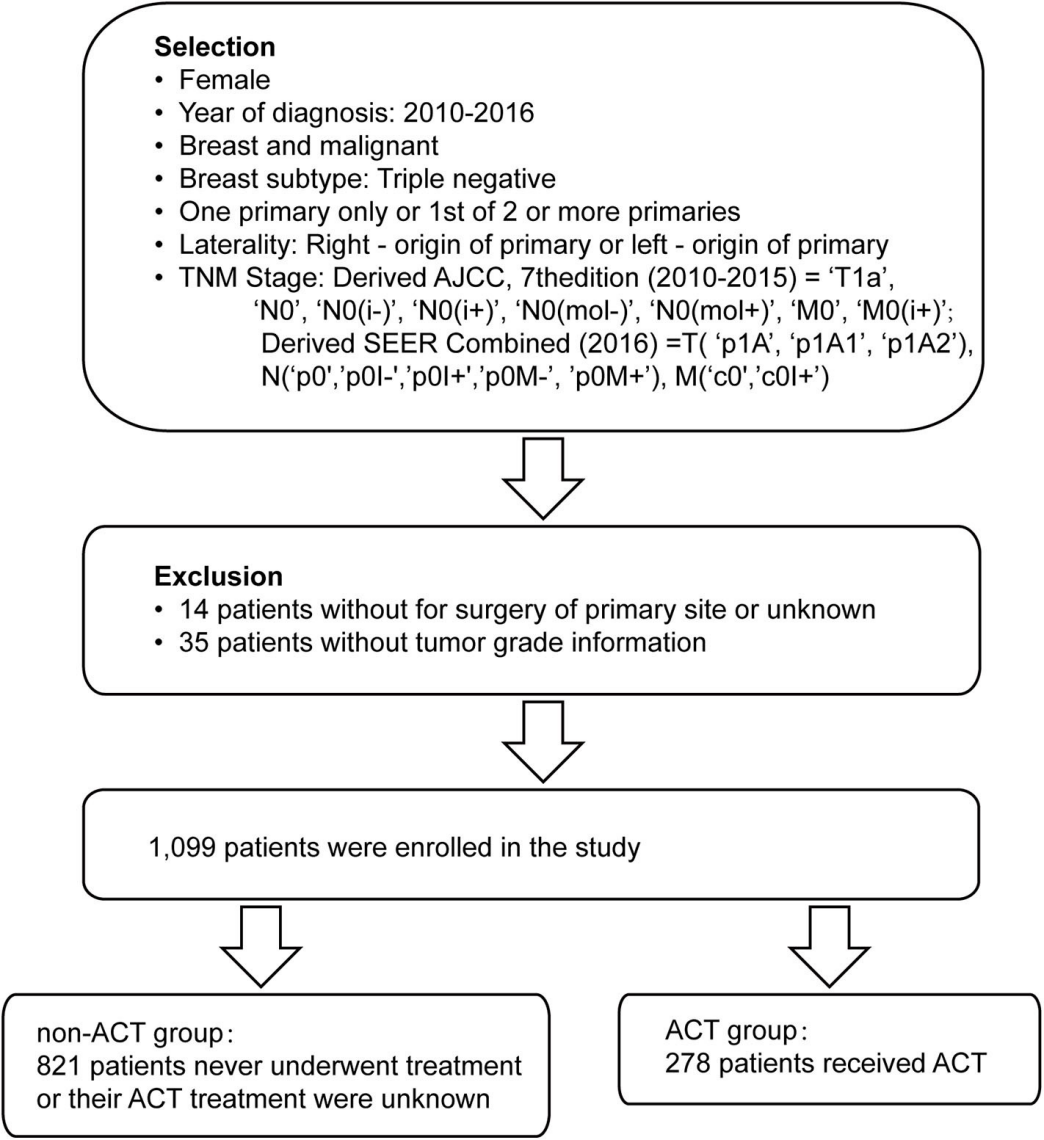
The selection and grouping of patients for the study.

Altogether, there were 1,099 cases that met the criterion of this study (Figure 1). Among them, 278 patients received ACT, and 821 patients never underwent treatment or their ACT treatment was unknown. Median follow-up for these patients was 36 months. Patients with ACT tended to be younger than the group without ACT (<50 years old, 29.5 vs. 10.8%, *p* < 0.001). The higher the histological grade, the higher the percentage of patients who received ACT (grade I, 1.4 vs. 9.4%; grade II, 23.4 vs. 44.2%; grade III+IV, 75.2 vs. 46.4%, *p* < 0.001). In addition, a higher percentage of patients who received chemotherapy chose mastectomy (BCS, 60.4 vs. 68.0%; mastectomy, 39.6 vs. 32.0%, *p* = 0.022). No remarkable differences were observed between patients from the two groups in terms of race, marital status, laterality, or radiation therapy (Table 1). Considering the different distribution of several important prognostic indicators, we conducted a 1:1 (with ACT; without ACT) matched case-control analysis utilizing a PSM method, and 255 paired patients were finally identified (Table 1).

**Table 1:** Demographic and clinical characteristics of the study population.

**Characteristics**	**Before PSM^a^**		**Chi-square *p***	**After PSM**		**Chi-square *p***
	**No ACT^b^*N* (%)**	**ACT *N* (%)**		**No ACT *N* (%)**	**ACT *N* (%)**	
Median follow-up (months) (IQR^c^)	37 (18–57)	35 (19–57)		41 (25–61)	37 (20.5–57.5)	
Age (years old)			<0.001			1.000
<50	89 (10.8)	82 (29.5)		60 (23.5)	60 (23.5)	
≥50	732 (89.2)	196 (70.5)		195 (76.5)	195(76.5)	
Race			0.445			0.973
White	623 (75.9)	202 (72.7)		191 (74.9)	189 (74.1)	
Black	119 (14.5)	49 (17.6)		44 (17.3)	46 (18.0)	
Others	79 (9.6)	27 (9.7)		20 (7.8)	20 (7.8)	
Marital status			0.188			0.856
Married	474 (57.7)	173 (62.2)		155 (60.8)	153 (60.0)	
Not married	347 (42.3)	105 (37.8)		100 (39.2)	102 (40.0)	
Laterality			0.794			0.535
Left	406 (49.5)	140 (50.4)		127 (49.8)	120 (47.1)	
Right	415 (50.5)	138 (49.6)		128 (50.2)	135 (52.9)	
Grade			<0.001			0.930
I	77 (9.4)	4 (1.4)		3 (1.2)	4 (1.6)	
II	363 (44.2)	65 (23.4)		65 (25.5)	65 (25.5)	
III+IV	381 (46.4)	209 (75.2)		187 (73.3)	186 (72.9)	
Surgery type			0.022			0.522
BCS^d^	558 (68.0)	168 (60.4)		156 (61.2)	163 (63.9)	
Mastectomy	263 (32.0)	110 (39.6)		92 (38.8)	92 (36.1)	
Radiotherapy			0.610			0.371
No	343 (41.8)	121 (43.5)		115 (45.1)	105 (41.2)	
Yes	478 (58.2)	157 (56.5)		140 (54.9)	150 (58.8)	

a *Propensity score matching*.

b *Adjuvant chemotherapy*.

c *Interquartile range*.

d *Breast-conserving surgery*.

### Prognostic Factors Analysis

The multivariate Cox proportional hazards regression model was applied to study the survival outcome–related factors. As shown in Table 2, before PSM, BCSS showed no statistically significant connection with age, race, marital status, histological grade, chemotherapy, type of surgery, or radiotherapy. The type of surgery was an independent prognostic factor after PSM. Patients with mastectomy seem to suffer a worse BCSS than patients who received BCS [hazard ratio (HR), 18.753; 95% confidence interval (CI), 1.195–294.292; *p* = 0.037].

**Table 2:** Multivariate Cox proportional hazard model for assessing outcome-related factors of breast cancer–specific survival.

**Variable**	**Before PSM^a^**		**After PSM**	
	**HR^b^ (95% CI^c^)**	**Cox *p***	**HR (95% CI)**	**Cox *p***
**Age (years)**				
<50	1		1	
≥50	0.697 (0.184–2.646)	0.596	0.336 (0.064–1.762)	0.197
**Race**				
White	1		1	
Black	0.775 (0.166–3.614)	0.764	0.874 (0.088–8.685)	0.908
Others	0.644 (0.082–5.084)	0.677	2.412 (0.255–22.806)	0.442
**Marital status**				
Married	1		1	
Not married	2.771 (0.903–8.504)	0.075	3.582 (0.591–21.698)	0.165
**Grade**				
I + II	1		1	
III + IV	0.975 (0.330–2.875)	0.963	1.400 (0.226–8.671)	0.718
**Chemotherapy**				
No	1		1	
Yes	0.760 (0.200–2.892)	0.687	1.182 (0.235–5.957)	0.839
**Surgery type**				
BCS^d^	1		1	
Mastectomy	2.265(0.444–11.568)	0.326	18.753 (1.195–294.292)	0.037
**Radiotherapy**				
No	1		1	
Yes	0.579 (0.103–3.247)	0.535	4.237 (0.464–38.711)	0.201

a *Propensity score matching*.

b *Hazard ratio*.

c *Confidence interval*.

d *Breast-conserving surgery*.

Multivariate analysis of OS indicates that patients who were unmarried (HR, 2.410; 95% CI, 1.291–4.498; *p* = 0.006) or did not have radiotherapy (HR, 0.357; 95% CI, 0.163–0.781; *p* = 0.010) might have worse survival before PSM evaluation. However, after analysis was adjusted with variables that were reported to be associated with breast cancer prognosis, no independent prognostic factors for OS were found (Table 3).

**Table 3:** Multivariate Cox proportional hazard model for assessing outcome-related factors of overall survival.

**Variable**	**Before PSM^a^**		**After PSM**	
	**HR^b^ (95% CI^c^)**	**Cox *p***	**HR (95% CI)**	**Cox *p***
**Age (years old)**				
<50	1		1	
≥50	2.071 (0.625–6.862)	0.234	1.481 (0.413–5.318)	0.547
**Race**				
White	1		1	
Black	1.018 (0.462–2.240)	0.966	0.686 (0.145–3.241)	0.634
Others	0.702 (0.212–2.325)	0.563	1.390 (0.287–6.722)	0.683
**Marital status**				
Married	1		1	
Not married	2.410 (1.291–4.498)	0.006	1.662 (0.605–4.566)	0.325
**Grade**				
I + II	1		1	
III + IV	0.840 (0.458–1.542)	0.574	0.693 (0.242–1.985)	0.494
**Chemotherapy**				
No	1		1	
Yes	0.583 (0.239–1.422)	0.236	0.711 (0.257–1.969)	0.512
**Surgery type**				
BCS^d^	1		1	
Mastectomy	0.742 (0.343–1.607)	0.449	0.691 (0.162–2.943)	0.617
**Radiotherapy**				
No	1		1	
Yes	0.357 (0.163–0.781)	0.010	0.281 (0.062–1.277)	0.100

a *Propensity score matching*.

b *Hazard ratio*.

c *Confidence interval*.

d *Breast-conserving surgery*.

### Comparison of BCSS and OS Between Patients With Adjuvant Chemotherapy and Without Chemotherapy

**Figure 2:**
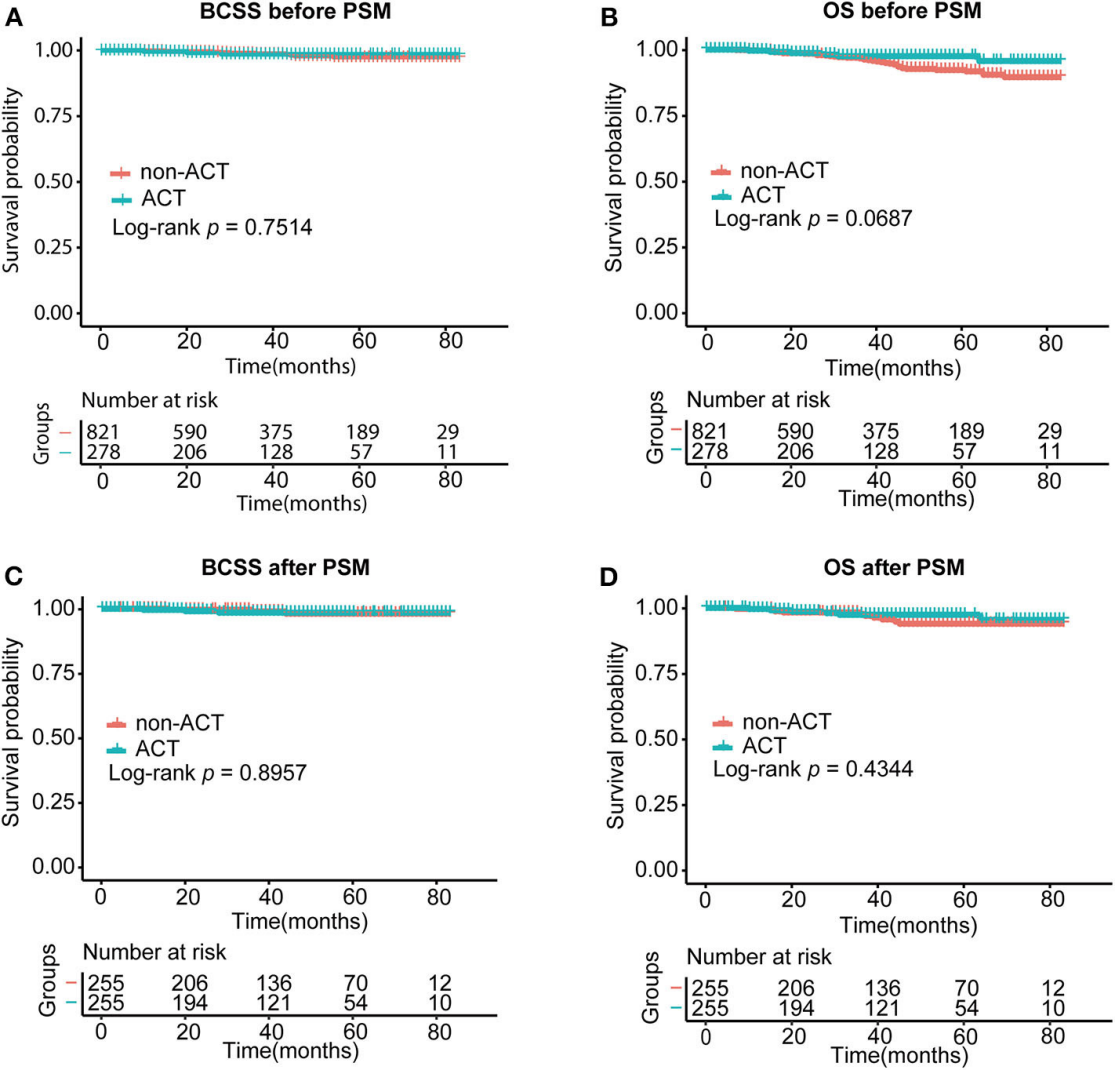
Survival curves of the two groups of patients with BCSS **(A)** and OS **(B)** before PSM and BCSS **(C)** and OS **(D)** after PSM. ACT, adjuvant chemotherapy; BCSS, breast cancer–specific survival; OS, overall survival. The log-rank test was performed to compare the BCSS and OS rates of the two group patients.

We used a Kaplan–Meier plot and log-rank tests to describe and compare the survival curves of the two groups of patients with BCSS and OS (Figures 2A,B). As stated, patients with and without ACT had similar BCSS (*p* = 0.7514) and OS (*p* = 0.0687). The 3-year cumulative survival probability of BCSS for the group with ACT was 98.5% and was 98.7% in the no-ACT group. A similar outcome was observed in 5-year cumulative survival probability of BCSS (98.5 vs. 97.4%). As for OS, 3-year cumulative survival probabilities were 97.5% (with ACT) and 96.7% (without ACT). However, 5-year cumulative survival probability of OS in patients without ACT reduced to 92.2%, and that in the ACT group was 97.5%.

After PSM, we saw no superior prognostic value with the use of adjuvant chemotherapy with regard to BCSS or OS (*p* = 0.8957 for BCSS and *p* = 0.4344 for OS) (Figures 2C,D). A similar 5-year cumulative survival probability for BCSS was found for the two groups (ACT, 98.5% vs. no ACT, 98.1%). Patients with ACT were related to better OS than those without ACT with the 5-year cumulative survival probability of BCSS reaching 97.3 vs. 93.9%. But the discrepancy was without statistical significance.

### Survival Prognostic Factors Associated With ACT

To distinguish the patient benefit from ACT, we stratified the patients according to their age, race, marital status, histological grade, surgical procedure, and radiotherapy after PSM. The univariate Cox proportional hazards regression model was used to compare the BCSS and OS between the two studied groups. As demonstrated in Table 4, no factors included in the analysis were associated with the survival of patients who received chemotherapy.

**Table 4:** Cox proportional hazard regression model of BCSS and OS comparing two studied groups, stratified by clinical variables after PSM.

**Variables**	**Without adjuvant chemotherapy vs. adjuvant chemotherapy^a^**			
	**BCSS**		**OS**	
	**HR^b^ (95% CI^c^)**	**Cox *p***	**HR (95% CI)**	**Cox *p***
**Age (years old)**				
<50	1.819 (0.164–20.129)	0.626	1.819 (0.164–20.129)	0.626
≥50	0.579 (0.052–6.382)	0.655	0.522 (0.161–1.695)	0.279
**Race**				
White	0.371 (0.039–3.568)	0.391	0.367 (0.099–1.355)	0.132
Black	63.819 (0.000–6.149E8)	0.612	1.195 (0.074–19.377)	0.900
Others	70.475 (0.000–6.90E8)	0.604	69.848 (0.001–6.114E6)	0.465
**Marital status**				
Married	1.097 (0.069–17.565)	0.948	0.360 (0.073–1.786)	0.211
Not married	1.092 (0.153–7.766)	0.930	1.124 (0.281–4.500)	0.869
**Grade**				
I + II	1.102 (0.069–17.625)	0.945	1.122 (0.226–5.559)	0.888
III + IV	1.086 (0.153–7.714)	0.934	0.477 (0.123–1.848)	0.284
**Surgery type**				
BCS^d^	0.017 (0.000–1.742E5)	0.622	0.421 (0.082–2.169)	0.301
Mastectomy	1.832 (0.305–10.990)	0.508	0.980 (0.263–3.653)	0.976
**Radiotherapy**				
No	3.583 (0.372–34.481)	0.269	0.671 (0.196–2.294)	0.525
Yes	0.016 (0.000–1,437.674)	0.479	0.676 (0.113–4.050)	0.668

a *Group without adjuvant chemotherapy is reference*.

b *Hazard ratio*.

c *Confidence interval*.

d *Breast-conserving surgery*.

## Discussion

A retrospective study enrolling 51,246 cases of T1a, bN0M0 breast cancer demonstrated that the OS proportion at 5 and 10 years was 90.2 and 75.9%, the 5- and 10-year BCSS proportion was about 98 and 96% (17). However, there are high risks of recurrence or distant metastasis in some patients with T1a, b tumors (17, 18). A multicenter retrospective cohort study (19) found that T1a tumors had a worse prognosis than T1b or even T1c tumors when considering RFS or DRFS, implying that patients with T1a tumors usually lacked enough treatment. Patients with T1a tumors exhibited several unfavorable prognostic factors and unexpected differences from patients with T1b or T1c tumors, especially those with the HR-negative, HER2-overexpression and triple-negative subtypes. Although ACT or trastuzumab or endocrine therapy is not strongly advised for T1a tumor patients according to guidelines, it is true that small tumor size itself is not a sufficient prognosticator (20). Considering these factors, the treatment regimens containing ACT should be prescribed in accordance with the risk associated with the different subtypes and their respective biological characteristics.

With regard to TNBC tumors of small size, some studies have indicated that a fraction of patients might benefit from adjuvant chemotherapy (11, 16). Zaida Morante et al. evaluated a T1N0-TNBC-patient cohort diagnosed between 2000 and 2014 at a single institution and found a better outcome in patients treated with ACT (21). However, most experts oppose routine ACT for pT1a pN0 TNBC (22) because the evidence of an advantage for ACT in these patients is inadequate. There have been other previous retrospective studies that discuss the impact of chemotherapy on subcentimetric TNBC (14, 15). They focused on the T1a, b subgroup; regretfully, the cases of T1a TNBC are rare. In addition, T1aN0M0 TNBC cases have been excluded from previous clinical trials in normal conditions, and it is unrealistic and difficult to conduct a randomized clinical trial including these few patients with or without ACT.

Our aim was to investigate the effect of adjuvant chemotherapy on the survival of T1aN0M0 TNBC patients. We analyzed patients of the cohort utilizing population-based SEER data and found that the BCSS and OS is not significantly different between groups of patients with and without ACT. After having matched and considered confounding factors, including age, race, marital status, histological grade, surgery type, and radiotherapy, no striking difference in survival outcome was observed between the two groups of patients. Our result is in accordance with a previous study that found patients with subcentimetric tumors, including T1a and T1b, node-negative TNBC, receiving ACT did not derive a significant DFS or distance metastasis free survival (DMFS) benefit (15). Furthermore, in our study, the 3- and 5-year BCSS of the patients in the ACT group are both 98.5% after PSM with the value up to 98.9 and 98.1%, respectively, in the opposite group. The favorable survival is probably the reason that T1a N0 TNBC patients did not show a prominent benefit from adjuvant chemotherapy.

Our study discovered that T1aN0M0 TNBC patients with younger age or higher histological grade were more likely to receive ACT. A previous study demonstrates that older patients have a better prognosis but worse tolerance toward ACT than younger patients (23). And being younger is regarded as a classical poor prognostic factor that is related to loco-regional relapse, BCSS, and OS (24) with small breast cancer tumors (25). In addition, the histological grade of the tumor is also a recognized prognostic indicator connected with survival (26, 27). Therefore, young age and high histological grade seem to be the usual reference factors when considering ACT for T1a TNBC patients. Our research indicates that, after stratification analysis according to the traditional risk factors, no distinct prognostic factors for survival associated with ACT were found in T1aN0M0 TNBC patients. Some previous studies suggest that some known prognostic factors are not applicable for TNBC. For instance, Ki-67 and the grade did not show any prognostic value in TNBC according to a study by Schmidt et al. (27). Rakha et al. (28) finds that the patients' age, tumor size, and androgen receptor expression were not of significant value in determining the prognosis in a N0TNBC group. Through a series of analyses, we draw the conclusion that T1aN0M0 TNBC patients treated with ACT have similar survival outcomes compared to those who did not receive ACT, and this result is not influenced by classical prognostic factors.

In matched groups, we find that type of surgery is an independent prognostic factor of BCSS. Patients with BCS have a better BCSS than patients who had a mastectomy. This conclusion was consistent with previous reports (29, 30). Compared with mastectomy, early stage TNBC patients who underwent BCS have a lower risk of loco-regional recurrence and of developing distant metastasis.

A characteristic of our study is that it is the first study evaluating adjuvant chemotherapy efficacy in T1aN0M0 TNBC patients using a large population data set. Moreover, we reduced the effect of confounding factors by enforcing PSM. There are, however, several limitations to the study. First, the median follow-up time of 36 months was not enough with the information about adjuvant chemotherapy and HER2 status in the SEER data available only from 2010 to 2016. Further outcomes of the cohort patients should be considered in the future. Second, the details of the adjuvant chemotherapy regimens and the duration of the patients' disease were unknown so that we cannot determine the different survival outcomes of different treatments. Finally, further analysis cannot be carried out because of a lack of certain prognostic indicators, such as Ki-67, BRCA mutations, and androgen receptor expression.

Collectively, our study indicates no strong association between adjuvant chemotherapy and better survival in T1aN0M0 TNBC. In addition, young age, race, marital status, histological grade, and radiation do not seemingly predict a survival benefit in these cohorts. We provide a clue for the treatment strategies of ACT in T1aN0M0 TNBC patients. However, therapeutic strategies for T1aN0M0 TNBC patients need more attention and further research.

## Data Availability Statement

Publicly available datasets were analyzed in this study. This data can be found at: https://seer.cancer.gov.

## Ethics Statement

This study was approved by ethics committee of Affiliated Union Hospital, Fujian Medical University. The data in the SEER database do not require informed patient consent because cancer is a disease reported by every state of the United States.

## Author Contributions

W-FF, Q-XC, and C-GS designed the study. W-FF, Q-XC, and X-XW obtained and analyzed the data. W-FF, Q-XC, JZ, and C-GS unscrambled the statistical result. W-FF and Q-XC wrote the manuscript. All authors reviewed and modified the manuscript.

## Conflict of Interest

The authors declare that the research was conducted in the absence of any commercial or financial relationships that could be construed as a potential conflict of interest.
